# Idiopathic Granulomatous Mastitis: Is It Still a Mysterious Entity?

**DOI:** 10.7759/cureus.81817

**Published:** 2025-04-07

**Authors:** Lamees Yaghan, Abdulla I Mohamed, Rami Yaghan

**Affiliations:** 1 Medical Skills and Simulation Center, Arabian Gulf University, Manama, BHR; 2 Department of Surgery, Arabian Gulf University, Manama, BHR

**Keywords:** classification, granulomatous lobular mastitis, idiopathic granulomatous mastitis, multidisciplinary team, treatment

## Abstract

Idiopathic granulomatous mastitis (IGM) is stereotypically described as a rare, mysterious breast disorder that is difficult to diagnose and treat. Initial literature about IGM consisted of case reports and retrospective case series. Understandably, this led to arbitrary, and occasionally contradictory, surgical and medical treatment approaches.

Over the last two decades, IGM has markedly departed from its classical description. It is no longer that rare disease and the spectrum of clinical presentation has widely expanded, both locally and systematically. In addition, a relatively good number of recent multicenter, meta-analysis, systematic reviews, and consensus reports about IGM have become available.

The advancements in the diagnostic techniques of IGM and the growing knowledge about IGM treatment options no longer justify the routine labeling of IGM as a mysterious entity. The preponderance of evidence is now in support of complementary, rather than contradictory, surgical and systemic immunosuppressive treatment.

Patients with IGM are better treated under the care of a multidisciplinary team. This will facilitate personalizing the treatment according to the needs of each patient with the minimum possible morbidity. There is a need for a comprehensive classification system for IGM that reflects the clinical variants, the radiological patterns, and the pathological details of IGM. Such a classification will provide useful hints about the treatment, the likelihood of the recurrence, and the expected natural history of IGM.

## Editorial

The classical description of idiopathic granulomatous mastitis 

Idiopathic granulomatous mastitis (IGM), also known as granulomatous lobular mastitis, was first described by Kessler and Wolloch in 1972 [1,2]. It is a form of non-caseating granulomatous breast disorder of obscure etiology [1,2]. Classically, IGM was described as a very rare disease occurring exclusively in parous young women. A hard breast mass that mimics breast carcinoma clinically, radiologically, and even cytologically was the usual presenting feature, to the extent that few patients underwent unnecessary mastectomies [3]. The diagnosis of IGM was a histological surprise after an excisional biopsy for what was assumed to be breast carcinoma. Surgical excision was the only adopted treatment during the first two decades after 1972. The recurrence rate of IGM was, and is still, high with an overall estimated rate of 17.18% [4].

The above features led to the still ongoing stereotypical description of IGM as a mysterious entity that is difficult to diagnose and treat.

The changing face of idiopathic granulomatous mastitis

IGM has departed largely from its classical description. The incidence of IGM is rapidly increasing with a growing global awareness about its potentially serious impact on young women's health [5]. There has been a marked worldwide rise in the number of case series and other categories of publications about IGM over the last two decades. During the last four years, 326 and 256 new publications about IGM were indexed in Scopus and PubMed, respectively. It is worth mentioning here that IGM is no longer restricted to the female gender. Until the year 2020, 13 IGM cases were reported in men [6].

The spectrum of clinical presentations of IGM has markedly expanded compared to the original description. It is no longer restricted to hard breast lumps. Patients might also present with breast abscesses, subacute fungating breast lesions, or chronic mammary fistulas and sinuses [1]. A wide range of associated extra-mammary presentations are occasionally reported including erythema nodosum, arthralgias, arthritis, oligo- arthritis, and episcleritis [1].

The diagnosis of IGM should no longer be a histological surprise after excisional biopsies. The presence of a sizable hard breast mass of a short duration prior to presentation, the presence of variable degrees of breast pain and local inflammation, and a high index of suspicion increase the likelihood of early clinical suspicion of IGM. The vast majority of IGM cases are now diagnosed on the basis of true cut biopsies allowing better patient counseling and increasing the chance of preoperative corticosteroid treatment in properly selected cases [1,7].

More clues about the diagnosis of IGM by ultrasound have now been identified [1,2]. The most common ultrasound finding is a hypoechoic mass with a characteristic tubular hypoechoic extension [1]. Doppler imaging shows increased internal blood flow within the lesion and the surrounding parenchyma [1]. Few new techniques were suggested to increase the specificity of ultrasound in diagnosing IGM. Examples include the combination of strain elastography with B-mode ultrasound and acoustic radiation force impulse imaging [1]. The role of MRI in the diagnosis and follow-up of IGM is gaining more interest [2].

Many authors are still stereotypically overemphasizing the absence of an established treatment for IGM and that such treatment depends solely on the discretion of the treating clinician. This was understandable in the first few decades after recognition of IGM in 1972, because the initial literature consisted of case reports and limited retrospective case series. In contrast to this, a relatively good number of multicenter studies, meta-analysis studies, consensus reports, and systematic reviews about IGM are available now [2,8-11]. The preponderance of evidence is now in support of a complementary, rather than a contradictory, balance between wide local surgical excision and systemic immunosuppressive corticosteroid treatment [8-10].

In recurrent or resistant IGM, adding methotrexate or azathioprine seems to be beneficial [1,12].

Most investigators consider IGM as a local immune response that involves both humoral and cell-mediated immunity [1]. The triggering antigen could be an unidentified infectious agent or a glandular secretory antigen [3]. Associations with parity, lactation, and pregnancy are universal findings [1,2].

Authors' insights

A patient with IGM is better treated under the care of a multidisciplinary team including a breast surgeon, an internist (or immunologist, rheumatologist, oncologist), a radiologist, and a pathologist. A multidisciplinary approach is expected to reduce the rate of initial erroneous clinical impression of carcinoma and personalize the treatment according to the needs of each patient with minimum possible morbidity. A combination of wide local surgical excision and prednisolone is the mainstay treatment [5,8-10]. Issues related to treatment sequence, duration of medical treatment, extent of surgical resection, individual patients’ needs, and follow-up period are best tackled under the multidisciplinary umbrella.

**Figure 1:**
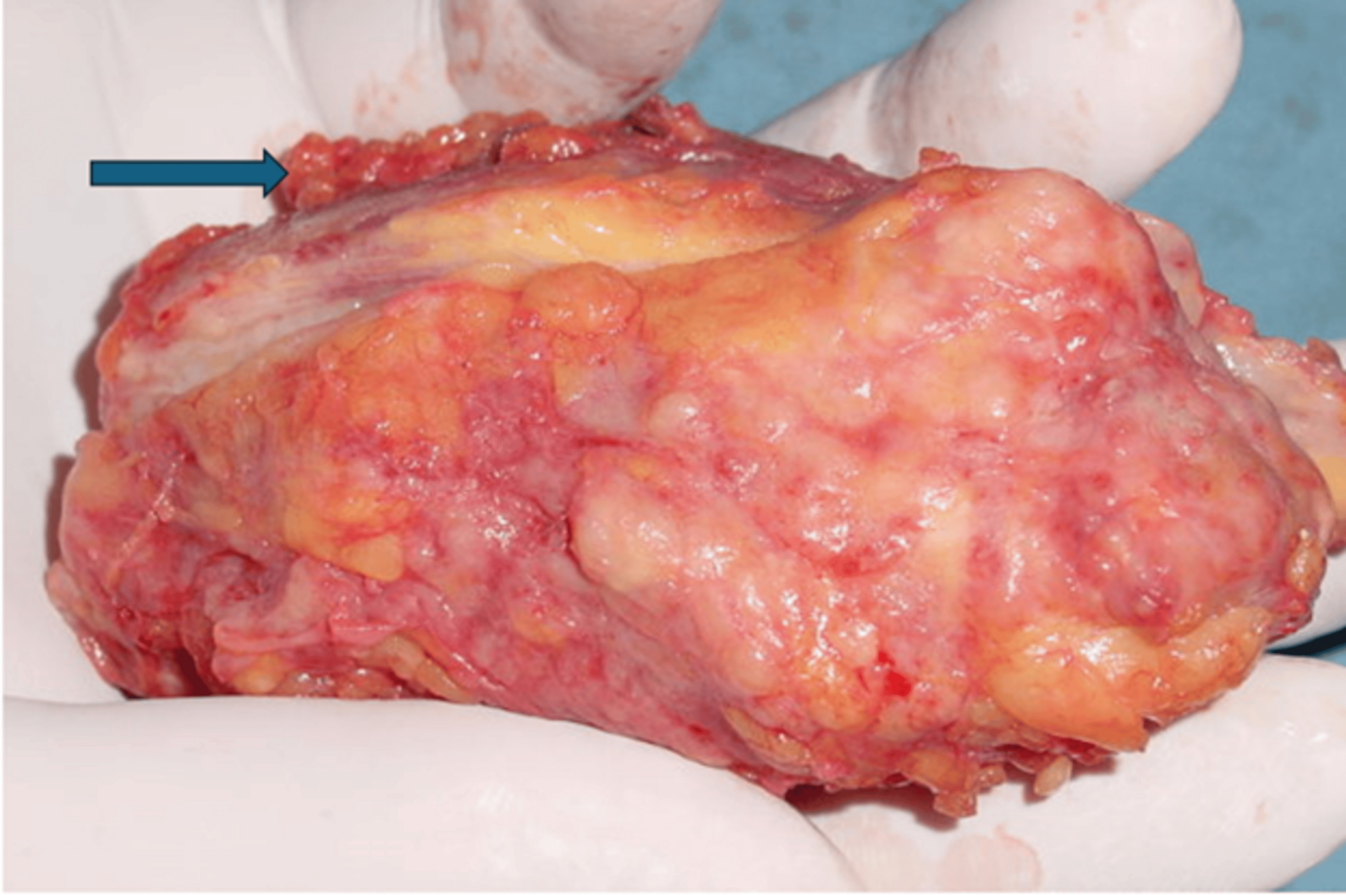
Gross appearance of the classical variant of idiopathic granulomatous mastitis. This 8×3 cm mass was excised from the right breast of a 40-year-old female patient. The mass was painless and hard with no gross inflammatory signs. The outer surface of the mass (arrow) is often adherent and difficult to separate from the normal surrounding breast tissue. Source: Image from Rami Jalal Yaghan's case files.

**Figure 2:**
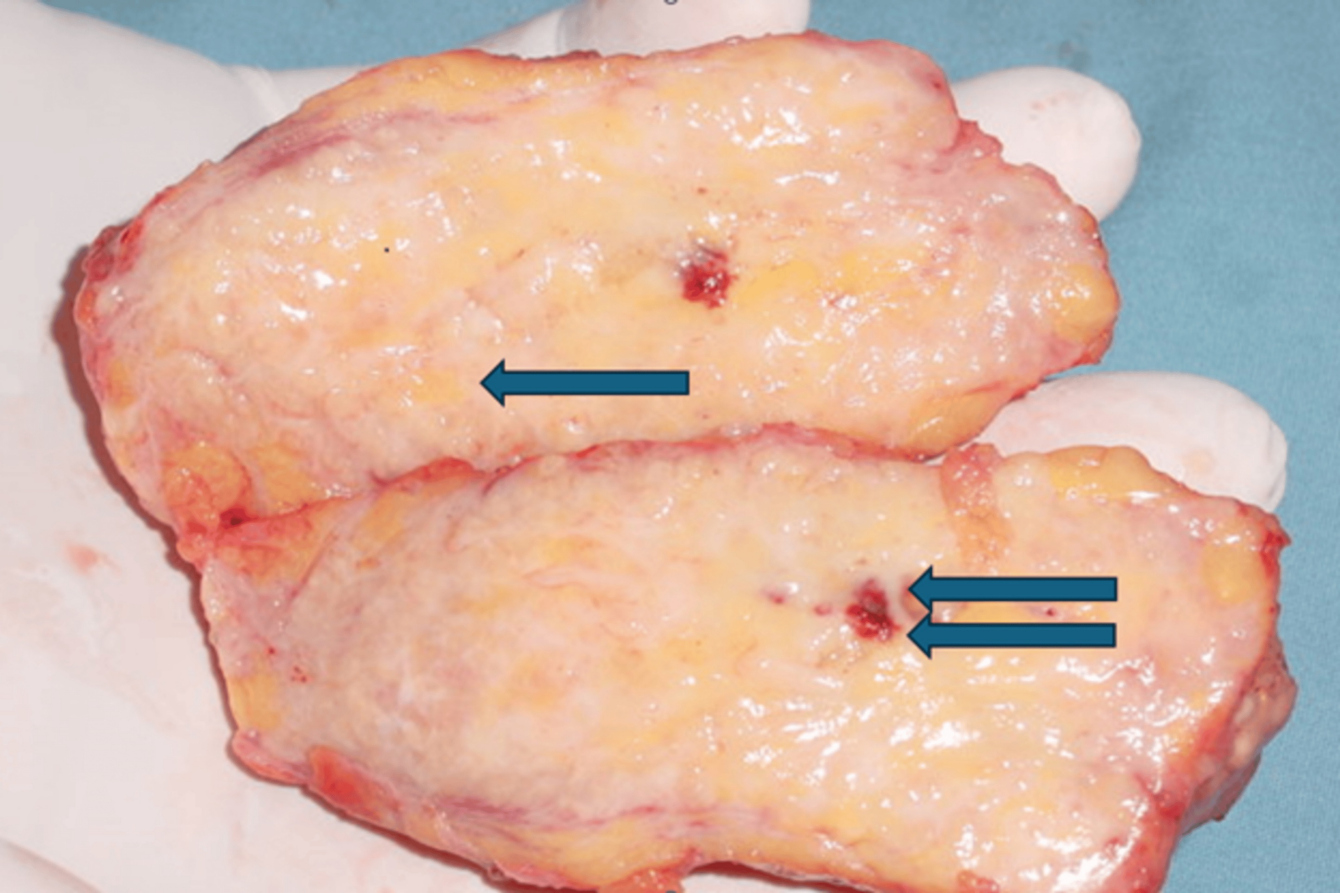
Cross-sectional appearance of the classical variant of idiopathic granulomatous mastitis. The glistening yellow patches (single arrow) and the small hemorrhagic areas (double arrows) are characteristic features of the classical variant of idiopathic granulomatous mastitis. This is the same mass seen in Figure 1. Source: Image from Rami Jalal Yaghan's case files.

The still appearing sharp radical statements that IGM is strictly a medical disease are not consistent with real clinical practice. How can patients with associated abscesses or chronic fistulas be solely treated by corticosteroids? In addition, the use of corticosteroids might be limited in pregnant, diabetic, or lactating women. Some women might also be reluctant to receive steroids in view of the potential adverse events. This becomes more relevant in the absence of predictors of treatment durations and doses. By the same token, a sharp statement that surgery is the monotherapy of choice is refuted by the current literature. We believe that the only fraction of patients who might be an exception are those presenting with a small or a moderately sized mass in the absence of any gross inflammatory signs (Figure 1 and Figure 2). Wide local excision alone in these patients seems to produce rapid disease control with a relatively low rate of recurrence [13]. Actually, this is the classical variant of IGM described 53 years ago. A short preoperative course of prednisolone can be very effective in this subgroup of IGM patients when presenting with large masses. This will reduce the extent of surgery with a better cosmetic outcome.

Surgery is an indispensable treatment component of extensive suppurative types of IGM. Such patients tend to have a more protracted disease course and more recurrences. In a few scattered reports, this has been misinterpreted as evidence that patients having surgery as part of their treatment have worse treatment outcome. Actually, this group of IGM patients had worse results because of their severe disease, and not because surgery was part of their treatment.

Despite the improving diagnostic radiological techniques, direct contact between clinicians and radiologists is of utmost importance in improving the radiological diagnostic accuracy of IGM.

There is a need for a comprehensive classification system for IGM. An ideal classification should reflect the various clinical presentations, radiological patterns, and pathological features of IGM. Such a classification will be expected to provide useful practical hints about the treatment, severity, and likelihood of recurrence of IGM. Two clinically based classifications with therapeutic implications were proposed, but a comprehensive classification incorporating the above variables is still lacking [11,13].

The natural history of IGM is still not well understood. Are we dealing with progressive stages from a hard mass to abscess formation that ends in chronic sinuses and fistulas, or are these distinct clinical variants to start with?

The risk factors associated with the recurrence of IGM are currently a major concern [4]. Modification of treatment modalities according to the presence or absence of these factors is another issue to be addressed. Some predictive models for recurrence were suggested but were limited by the small number of patients included. More research is needed in this area.

In a recent systematic review, intralesional corticosteroid was associated with a reduced recurrence rate, however, no studies included patient-reported outcomes [14].

**Figure 3:**
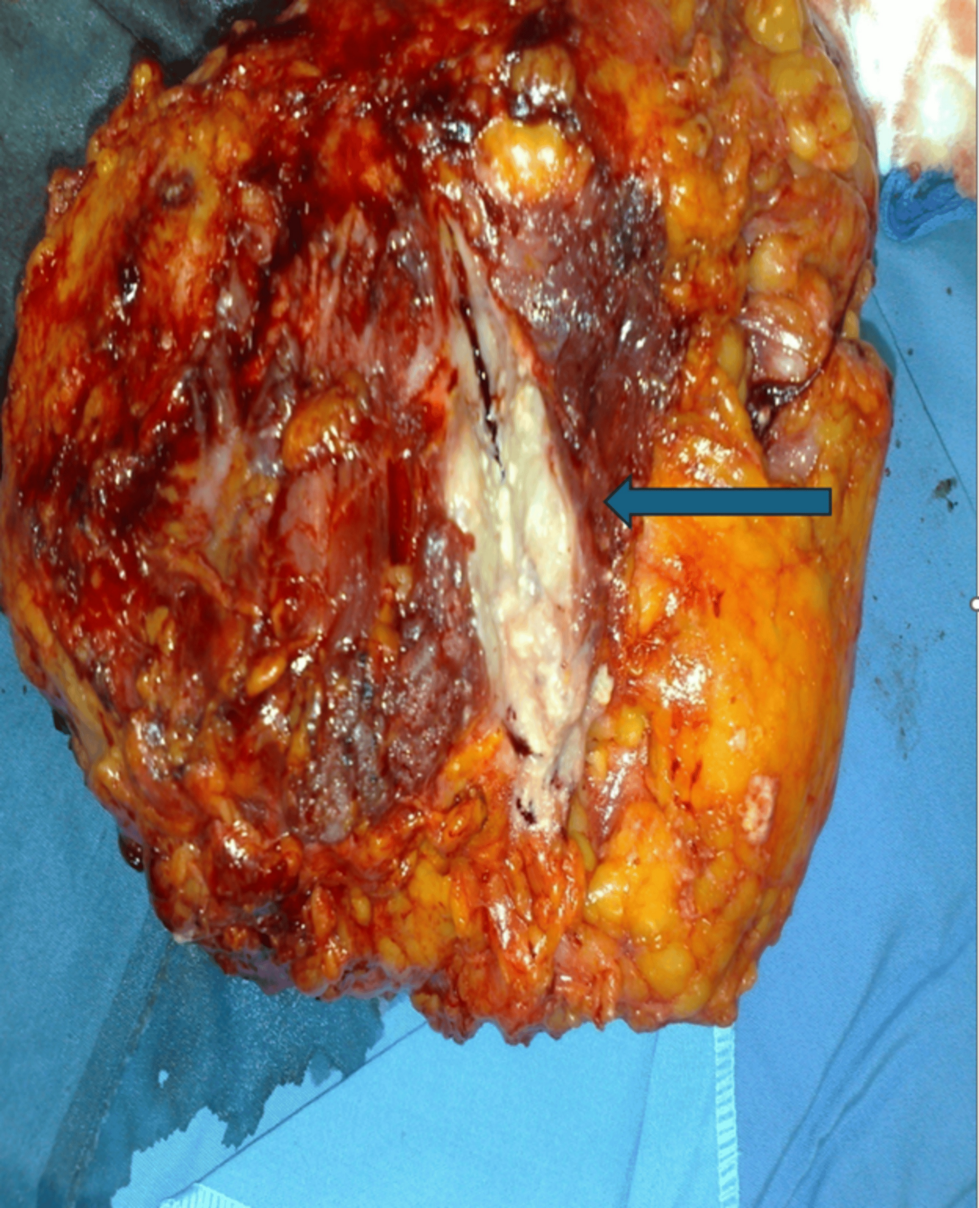
Idiopathic granulomatous mastitis presenting as a chronic abscess. This huge mass was excised from the left breast of a 35-year-old female patient. The mass was painful and firm. Note the thickened abscess wall with extensive fibrous tissue formation (arrow). The mass destructed the medial aspect of the breast. A combined treatment of surgical excision and prednisolone treatment resulted in permanent remission. Source: Image from Rami Jalal Yaghan's case files.

The isolated incidences of spontaneous resolution of IGM should be interpreted very cautiously. IGM can be very destructive, and few patients have lost their breasts because of inadequate treatment (Figure 3) [13].

Cystic neutrophilic granulomatous mastitis (CNGM) is a recently identified entity of IGM that might be related to bacterial infection, namely, Corynebacterium parakroppenstedtii and to a lesser extent some Staphylococcus species [1,5]. Should CNGM still be considered as a variant of IGM or, more logically, a secondary type of granulomatous mastitis?

Apparently, the growing knowledge about IGM, the advancements in diagnostic techniques, and the availability of a reasonable consensus about treatment approaches have largely abolished the mysteriousness label of IGM [2,8-10]. The still obscure etiology is an exception to this and is an ideal target for translational research.

Conclusion

IGM is departing from its stereotypical description as a very rare cancer-mimicking breast disorder that is difficult to diagnose and treat. Prevalence, gender distribution, clinical presentation, and diagnostic techniques have markedly expanded. Recent consensus, systematic review, and meta-analysis studies are in support of a complementary surgical and systemic immunosuppressive therapy. The authors call for a multidisciplinary approach in this regard. There is a clear need for a comprehensive classification system for IGM that provides diagnostic, therapeutic, and prognostic clues. The natural history of IGM is still not well understood.
